# Nano-inspired smart interfaces: fluidic interactivity and its impact on heat transfer

**DOI:** 10.1038/srep45323

**Published:** 2017-03-27

**Authors:** Beom Seok Kim, Byoung In Lee, Namkyu Lee, Geehong Choi, Thomas Gemming, Hyung Hee Cho

**Affiliations:** 1 IFW Dresden, P. O. Box 270116, 01171 Dresden, Germany; 2 Samsung Electronics Co., Ltd, Digital Appliance Business R&D team, Samsung-ro 129, 16677 Suwon, Korea; 3 Department of Mechanical Engineering, Yonsei University, Yonsei-ro 50, 03722 Seoul, Korea

## Abstract

Interface-inspired convection is a key heat transfer scheme for hot spot cooling and thermal energy transfer. An unavoidable trade-off of the convective heat transfer is pressure loss caused by fluidic resistance on an interface. To overcome this limitation, we uncover that nano-inspired interfaces can trigger a peculiar fluidic interactivity, which can pursue all the two sides of the coin: heat transfer and fluidic friction. We demonstrate the validity of a quasi-fin effect of Si-based nanostructures based on conductive capability of heat dissipation valid under the interactivity with fluidic viscous sublayer. The exclusive fluid-interface friction is achieved when the height of the nanostructures is much less than the thickness of the viscous sublayers in the turbulent regime. The strategic nanostructures show an enhancement of heat transfer coefficients in the wall jet region by more than 21% without any significant macroscale pressure loss under single-phase impinging jet. Nanostructures guaranteeing fluid access via an equivalent vacancy larger than the diffusive path length of viscid flow lead to local heat transfer enhancement of more than 13% at a stagnation point. Functional nanostructures will give shape to possible breakthroughs in heat transfer and its optimization can be pursued for engineered systems.

Cooling and thermal energy transfer technologies are essential to guarantee the reliability of many engineered devices by assuring local/overall thermal stability^1^,^2^. Promising cooling and energy transfer technologies are commonly based on individual or coupled forms of the fundamental schemes of thermal conduction, convection, and radiation^3^,^4^,^5^,^6^,^7^. In particular, convection, which is based on the interfacial interactions between solid surfaces and counteracting fluidic media transferring the energy, is one of the most powerful and pragmatic heat/energy dissipating mechanisms, with broad feasibility and applicability. As convection is based purely on the fluidic behavior of the energy transferring medium and its interaction at the interface, interfacial effects – with respect to morphology-induced fluidics – are a decisive factor for characterizing heat and mass transfer performance.

For the improvement of interface-dependent convection, recent approaches have focused on the peculiarities of novel nano-inspired interfacial structures^8^,^9^,^10^,^11^,^12^. Nanostructured surfaces have been adopted to improve convection based on their intrinsic rough morphologies and the consequent functionality for fluidic interactivity. Such nano-modulated morphologies obviously extend the interfacial area between a heat-dissipating surface and working fluids. The roughened morphology offers a prerequisite for strong capillary force, *i.e.*, hemi-wicking, especially for liquids^13^,^14^,^15^,^16^, and thus leads to favorable static hydrophilicity and dynamic refreshing towards the surface^17^,^18^,^19^,^20^,^21^,^22^. Based on these concepts, forested silicon nanowires and analogous vertically standing nanostructures have been adopted to enhance heat transfer. In boiling heat transfer, they could lead to extraordinary enhancement of critical heat flux and heat transfer coefficients^12^,^23^,^24^,^25^. The beneficial characteristics of the nano-inspired interface can also be emphasized for single-phase convection schemes, according to the advantageous aspects of fine morphology manipulations, and the possibility of controlling interactivity with a fluidic boundary layer formation under forced convection^26^,^27^,^28^,^29^.

Here, we demonstrate the merits of a Si-based nano-inspired interface for single-phase liquid (de-ionized (DI) water) impinging-jet cooling, particularized for concentrated heat-dissipation ability against a high thermal load. We uncover that the interactivity of the nanostructures with fluidic boundary layer development can trigger a peculiar breakthrough in heat transfer performance: the prevention of friction-induced pressure drop can be achieved by disturbance-attenuated interfacial structures wholly confined within the stable viscous sublayer domain along a convective fluidic flow. We discuss a design strategy of the functionalized interfaces that do not lead to any significant macroscale pressure-drop increase, which is a common trade-off in heat transfer by surface roughening. Moreover, the validity of a quasi-fin effect for conductive heat dissipation through Si-nanostructures is discussed. These functionalities of Si-nanostructures are highlighted under the convective impinging jet, involving locally concentrated fluidic momentum flow and sequential wall jet development in a confined channel. For demonstrations, we devised an array-type local temperature measuring sensor (Fig. 1a–c) with *in-situ* Si-nanostructures and checked the repetitiveness of experiments. In this study, two kinds of Si-nanostructures, synthesized by the top-down metal-assisted chemical etching method^30^,^31^,^32^,^33^,^34^, are employed: randomly dispersed vertical silicon nanowires (SiNWs, Fig. 1d), with an average height, diameter and distance of 7 μm, 150 nm and 300 nm, respectively, and regularly arranged silicon nanopillars (SiNPs, Fig. 1e) with a height of 2.5 μm and controlled diameter and pitch of 250 nm and 610 nm, respectively (See Supplementary Information for material preparations, experimental setup for impinging jet, procedure, data reduction, and uncertainty analysis).

## Results and Discussion

### Hydraulics and heat transfer characteristics in impinging-jet

Figure 2a and b show schematics of the experimental apparatus for a closed-loop channel and a test section for the installation of the local temperature measuring sensor with *in-situ* Si nanostructures, respectively. Impinging-jet, a pragmatic fluidic and heat transfer scheme, accompanies complicated hydraulic phenomena according to its regime: near the impinging area affected by the jet core and its viscous shedding as well as the wall-jet area downstream of fluids as shown in Fig. 2c. The effects of normalized channel height (*H*/*W*), as a principal factor characterizing the impinging jet and sequential wall jet flow, are presented in Fig. 3a. We can see overall heat transfer performance is highest when the channel is most confined by *H*/*W* = 2. By increasing *H*/*W*, heat transfer coefficient *h* gradually decreases because local wall jet flow is decelerated under an extended flow channel. The decrease of the overall heat transfer coefficient is up to 20%, according to the variation of *H*/*W* from 2 to 8. In the light of local characteristics near the stagnation point, a definite peak value can be monitored at *x*/*W* = 0.5 as the inlet (*i.e.*, jet) Reynolds number (*Re*_*jet*_) increases. This can be explained as the destabilizing effects caused by fluidic shear between the jet core, which is profiled along the nozzle neck, and the stagnant stream at the edge of the nozzle^35^,^36^,^37^,^38^. Herein, the straight fully developing region is sufficiently guaranteed for the jet, with a length 15 times the nozzle width (*W*) for a stable velocity profile at the nozzle exit, which has a cross-section of 3.0 mm × 12.0 mm (See Supplementary Information for the test section preparation). Particularly in the submerged jet, there is prominent shear-interaction between the injecting jet stream and a stagnant fluid. Vortex shedding occurs along the interface of the jet stream towards an impinging wall^37^,^39^,^40^,^41^. The vortex increasing turbulence intensity starts to be generated at the edge of the nozzle due to the shear between the jet core and the stagnant fluid surrounding the core. Within the viscous shedding region as illustrated in Fig. 2c, continuous vortex rings, so called the primary vortices, propagate from the edge of the jet nozzle towards an impinging wall. Thus heat transfer coefficients can have peculiar local peaks not on the stagnation point but on a spot sideways at *x*/*W* = 0.5^39^,^40^,^41^. As we can clearly see in Fig. 3b, the influence of this fluidic interaction becomes more dominant as *Re*_*jet*_ increases and thus the peak at *x*/*W* = 0.5 is reinforced with an increase in *Re*_*jet*_. This local peak is attenuated by lowering the fluidic momentum and no significant local deviation can be found when *Re*_*jet*_ is less than 1500 in laminar flow, which has more stable fluidic behavior around a jet core.

As the jet flows along the streamwise lateral direction, a fluidic boundary layer develops in the wall jet region. In all *H*/*W* and *Re*_*jet*_ cases, heat transfer coefficients commonly decrease along the streamwise direction. However, we can find a secondary local peak at a certain distance of *x*/*W* ≈ 4.0 because the local Reynolds number (*Re*_*x*_) increases while the wall jet stream after impingement flows along a wall jet region and induces a transition to a turbulent regime^42^. When the inlet jet Reynolds number is higher than 3000 in turbulent regime, the secondary peak is more clearly seen in the wall jet region. Herein, the geometric channel confinement is also of importance in characterizing this fluidic transition^39^. As shown in Fig. 3a, flow rate reinforcement no longer contributes to this transition if the channel is not confined, with a high *H*/*W* of 8. Otherwise, when the channels have *H*/*W* of less than 8 within the present test region, there exists a secondary peak in the flow transition in all *Re*_*jet*_ conditions (as presented in Fig. 3b for *H*/*W* ≈ 4).

Local characterization at a stagnation point under the impinging jet is desirable, as a design target, especially for local hot spot cooling^43^. Figure 4 shows stagnation Nusselt numbers and a regression analysis for a correlation function when *Re*_*jet*_ and the normalized height are changed simultaneously. Basically, the local Nusselt number (*Nu*) could be primarily dependent on *Re*_*jet*_ and the Prandtl number, reflecting the fluidic conditions and properties of the working fluid, respectively^43^. In case of this study, using DI water, it is shown that the stagnation Nusselt number (*Nu*_*0*_) increases with *Re*_*jet*_. Even though it is dependent on geometric variables involving the normalized height, its effect can be insensitively converged into a regression function of *Re*_*jet*_, as suggested in previous approaches^43^,^44^,^45^. In this study, the regression function is expressed as *Nu*_*0*_ = 2.54 *Re*_*jet*_^0.495^ and it has good validity compared with a previous model^45^, which considered the effects of *Re*_*jet*_ and geometric parameters simultaneously, within a ±10% deviation range.

### Strategic nano-interface employments and interactivity in a fluidic domain

The peculiarities of nanostructured surfaces have been highlighted for improving heat transfer performance^9^,^10^,^11^,^12^,^24^,^25^. In particular, forested silicon nanowires and analogous vertically standing nanostructures have been widely used to enhance heat transfer accompanying phase changes in working fluids and they can lead to extraordinary enhancements of critical heat flux and heat transfer coefficients^16^,^25^,^46^,^47^. Attractive characteristics in heat transfer applications include the fact that nanoscale structures can present an extremely rough interfacial morphology. This obviously extends the interfacial area between a heat-dissipating surface and working fluids. Especially for liquid, the roughened morphology does offer a strong capillary force, hemi-wicking^15^,^19^, and thus leads to favorable static hydrophilicity and dynamic wetting or refreshing towards the surface^19^,^20^. However, the benefits of interfacial structures in single-phase applications should be distinguished from those in boiling heat transfer because the mechanism of heat transfer is totally different from boiling accompanying phase changes. Herein, we can demonstrate advantageous aspects of fine nanostructures for single-phase impinging-jet applications.

Impinging-jet cooling is a powerful heat dissipating scheme according to the effects of concentrated jet-momentum and sequential convection characteristics^44^,^48^. Herein, the nano-inspired interfaces can be utilized to stimulate peculiar fluid-interface interactivity. The impacts of nanostructures can be highlighted through a comparison of the heat-dissipating efficiency of heat transfer coefficient versus that on an untreated surface, as shown in Fig. 5a. They do not have significant differences near the stagnation area. However, the heat transfer enhancement by SiNWs can be clearly observed after the impingement-governing area. The difference, *i.e.*, the improvement due to SiNWs, accumulates along the wall jet region and the enhancement increases gradually as the stream flows downward. The overall distribution of heat transfer coefficient along the flow direction has a similar pattern as *Re*_*jet*_ changes; the two local peaks are observed near the nozzle edge at *x*/*W* = 0.5 and the transition point at *x*/*W* ≈ 4 while the overall *h* tends to increase as *Re*_*jet*_ increases. In accordance with the effects of the Reynolds number, the nano-inspired enhancements can be seen in each case. At *x*/*W* = 6.0, the difference in local heat transfer coefficient reaches 29.8%, 29.2%, and 21.3% according to *Re*_*jet*_ of 1000, 3000, and 5000, respectively.

When we consider that heat transfer in the stagnation area is governed by the primary jet momentum rather than by an interface-inspired effect, an instinctive nanostructure re-design can be feasible in the stagnation region by guaranteeing the direct jet stream towards a target surface. Especially near a stagnant point, the jet flow must be exhausted as it gets close to an impinging wall; that is, fluidic dynamic energy is converted into total energy without apparent momentum-governing behavior of the fluid. In this confined stagnating area, local fluidic motions can be explained by diffusive- or viscid-dominant molecular movements^49^. The viscid-dominant molecular behavior is governed by intrinsic properties of a media, confining individual motions of the liquid molecules against an infinite diverging diffusion. Considering the factors of the liquid behavior, the characteristic length of the viscid-dominant behavior, *λ*_*ν*_ can be expressed as *ν*·*u*^−1^, where *ν* and *u* are the kinematic viscosity of a fluid and apparent velocity of a fluid, respectively. In case of the complete energy dissipation, local fluidic motions should be governed by diffusive- (or viscid-) dominant molecular physics where an apparent (or average) velocity of a fluid is almost null. The apparent velocity of a jet stream indicates the critical condition which has the highest portion of the dynamic energy of a fluid. As for the most conservative case, it is assumed that a critical bound can be featured by considering an initial momentum of jet; an approaching velocity of a fluid *u*_*jet*_ as a given condition confining a critical or maximum bound of its initial motion. Considering the access of the liquid or convective effects on the local point, as described schematically in Fig. 6a, interfacial structures on the impinging area should result in larger vacancy compared with *λ*_*ν*_, which is characterized by ~450 nm in the case of *Re*_*jet*_ = 5000. Figure 5b shows the effect of the strategic use of regularly arranged SiNPs, which have a uniform and larger pitch (*λ*_*NP*_) of 600 nm. As the length of the nanopillars is diminished by 64% (2.5 μm), which efficiently decreases aspect ratio of structures, relative to that of SiNWs, they are more favorable for guaranteeing jet access by preventing the conglomeration of nanostructures. In the case of SiNWs, which have a high aspect ratio of 46.7, they should be distributed densely and be readily conglomerated with each other at the tip due to van der Waals forces^22^,^50^. Although this is one of the advantageous aspects in order to reinforce catalytic ebullitions in boiling heat transfer, the conglomerated feature is just an obstacle to single-phase convection, especially in terms of the direct accessibility of the jet towards a stagnant point^51^. We can confirm that strategic SiNPs with a controlled characteristic length (*λ*_*NP*_* > λ*_*ν*_) are effectively enhancing the local heat transfer even at the stagnation point by 16.5% and 13.1% versus the untreated surface and SiNW surfaces which were densely distributed without the sufficient vacancy, respectively. These improvements could be acquired by allowing for the viscid access of a fluid on the impinging surface.

### Quasi-fin effects via functional SiNWs and SiNPs

Although the degree of the enhancement and local characteristics differ according to the manipulated structure types and their characteristic lengths, the heat transfer in the wall jet region can be improved drastically by nanostructure implementation as shown in Fig. 5a and b. The increased contact area and its impact on heat dissipation capability are important factors in positive effects of nanostructures on impinging jet heat transfer. The enhancement of consequent heat transfer can be demonstrated based on the quasi-fin effect, as illustrated in Fig. 6b. The quasi-fin effects of the fine interfacial structures can be stressed in terms of their conductive heat-dissipating capabilities^52^,^53^. The scale of the applied interfacial structure is not large enough to cause a large-scale vortex as so-called macroscale structures do. Moreover, the structures were completely immersed in the highly stable viscous sublayer region of the wall jet flow; *l*_*Si*_ ≪ *δ*_*vis*_ where *l*_*Si*_ and *δ*_*vis*_ are the length of applied nanostructures and thickness of a viscous sublayer, respectively. Although the nanostructures have very small and confined characteristic diameters, distances and lengths, conductive heat dissipation via these structures can be rather emerged when they are thoroughly immersed in this viscous sublayer. The feasibility of the conductive heat dissipation through the fin-like structures can be addressed as an effective heat transfer ratio (*HTR*), which is the ratio of heat transfer from a finite fin to that from an infinitely long fin, expressed as follows^54^:


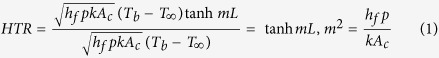


where *h*_*f*_, *p, k, A*_*c*_, *T*_*b*_, *T*_*∞*_, and *L* are the heat transfer coefficient at the exterior of the fin structure, perimeter of a cross section of a fin, thermal conductivity of a fin, cross sectional area of a fin, temperature at fin base, temperature of a surrounding medium, and length of a fin, respectively. Considering the morphology and properties of the synthesized nanostructures, it is assumed that the fin-like structure has a uniform cross-sectional area and homogeneous conductivity properties. Accounting for the feature of fin-like structures, the appropriate length of an equivalent fin is estimated by confining quantitative *HTR* values. The graphs presented in the inset of Fig. 5b are from 90% *HTR*. In the light of the characteristic dimensions of SiNWs and SiNPs, and their thermal conductivities (which are obviously less than those of bulk materials due to the confined phonon vibration in scaled-down nanostructures)^55^ we demonstrate that an appropriate length for a quasi-fin structure is sufficient in the regime of a few microns. Since the employed nanostructures have definitely low *Biot* numbers (*Bi* = *hd*/*k*), much less than 0.001 under a convection environment with impinging jets, the analytical approach defining a quasi-fin structure acquires its validity, neglecting radial temperature gradients in the nanostructures. According to the fundamentals of the plausible 1-D conduction approach, both vertically standing fin-like structures of SiNWs and SiNPs are effective in improving wall jet regime heat transfer. Considering the demonstrated peculiar impacts of nanostructures on local heat transfer characteristics at the jet-impinging dominant region, and in the wall jet dominant region we suggest there are design criteria reflecting the offset between spatial vacancy that guarantee direct fluidic accessibility favorable for the former region and the quasi-fin effect for the latter region, which requires a certain degree of extrusion as an equivalent fin structure. It was contradictory and ineffective to the local improvement especially at a stagnation point by prohibiting fluid access towards a heat transfer surface as the length increases. The demonstrations tell us that we can pursue optimal design criteria for local and overall enhancement considering this offset in dimensional approach in the interfacial structure design.

### Turbulent boundary layer: design of SiNWs/SiNPs for prevention of momentum loss

As one feasible design guideline for beneficial interfacial structures favorable to heat dissipation, we suggest a criterion concerning hydrodynamic boundary layer development on a heat transfer surface. A viscous sublayer, which is the first boundary contacting with a surface involved in a fluidic boundary layer, is important in determining interfacial effects on fluidic characteristics and consequent heat transfer ability. This layer is much thinner than that of the turbulent boundary layer and outer fluidic effects cannot readily reach the most stable viscous sublayer^56^,^57^,^58^. Thus, interfacial roughening by employing pin-fins and rib turbulators has been a feasible approach for enhancing convective heat transfer by directly disturbing the stagnant fluidic layer (*i.e.*, disturbing the turbulent boundary layer by *l*_*fin*_*  ≫ δ*_*TB*_, where *δ_TB_* is the thickness of the turbulent boundary layer). Improving convection is based on stimulating the interaction between a surface and a fluid, which can lead to higher turbulence intensity, whereas exclusive conductive heat dissipation can be alternatively pursued through the demonstrated fine quasi-fin structures, which do not cause fluidic disturbance as illustrated in Fig. 6b. Because the viscous sublayer is the most stable fluidic layer, the heat dissipation relies primarily on the conductive capability of the quasi-fins rather than convection. Although the quasi-fin structures are thoroughly immersed in the layer, they lead to an increase in the effective conductivity of the sublayer. This contributes to enhancing the role of dissipating thermal energy to a bulk boundary layer across the viscous sublayer and a following buffer layer. When we deal with a wall jet stream as a fluid flowing over a flat plate, we can estimate the quantitative thickness of the viscous sublayer (*δ_VSL_*) according to the law of the wall as follows^59^:


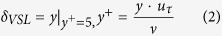


where *y, y*^*+*^, and *u*_*τ*_ are the vertical distance to the wall, dimensionless wall coordinate, and shear velocity, respectively. Considering a turbulent fluidic condition, the frictional coefficient can offer a clue for quantifying the shear velocity (*u*_*τ*_* = (τ*_*w*_/*ρ*)^1/2^, where *τ*_*w*_ and *ρ* are the wall shear stress and fluid density, respectively) as a function of *Re*_*x*_ and characteristic flow velocity (*u*_*∞*_) as *C*_*f*_* = *0.0594/*Re*_*x*_^−1/5^ = 2*τ*_*w*_/*ρu*_*∞*_^*2*^^59^. Figure 7 shows the evaluated thickness of the viscous sublayer according to *Re*_*jet*_ and dimensionless distance of *x*/*W* along a wall jet stream. *δ*_*VSL*_ in the turbulent regime is confirmed to be much larger than the height of the employed SiNWs and SiNPs of a few microns. This intuitively indicates that the nanostructures are not sufficient to cause the large-scale vortices or strong shear necessary for direct fluidic boundary disturbances. On the contrary, they can guarantee the stability of the viscous sublayer and the quasi-fin effects can be exclusively feasible for an effective conduction effect. That is, if the interfacial structures are thoroughly immersed in the viscous sublayer over a wall, with the characteristic lengths of the structures being much less than *δ_VSL_ (i.e., δ_VSL_* ≫ *l_fin_*), we can demonstrate that the quasi-fin effect can be reasonable, especially with respect to their conductive heat dissipation ability without peculiar convective-improving schemes. Additionally, the dimensional prerequisite guideline, validating the interfacial structures, can be highlighted to guarantee fluidic stability by minimizing the disturbance of fluidic friction, which is directly related to the pressure drop increase^27^,^28^,^60^. As indicated in the inset of Fig. 7, there are no remarkable pressure drop increases when we employ the nanostructure on a heat transfer surface from the stagnation point to the outlet of the wall jet flow. Although the use of nanostructures is finitely applied to a confined target area and is much less than the whole channel area, heat transfer enhancements at the target surface could be valuable for local cooling and efficient energy dissipation without any significant macroscopic pressure drop increase. For a novel local hot spot cooling scheme, we suggest that nano-inspired interfacial modification can be feasible with its fluidic and heat transfer advantages. Control of the characteristic length of the structures is important for characterizing the heat dissipation mode, especially at the wall jet region. The viscous sublayer thickness *δ*_*VSL*_ can be a critical guideline for determining the principal heat-dissipating mode and suggests a novel strategy for minimizing consequential pressure drop increase, which is seen commonly in convection-improving schemes by implementing macroscale surface roughening.

## Conclusions

Convection is a principal heat and energy transfer scheme for an essential hot spot cooling. Convection is purely dependent on interface characteristics; an avoidable trade-off in convective heat transfer improvement is interface-triggered fluidic resistance causing pressure loss. In this study, we revealed that the nano-inspired functional interface can be a promising candidate to take two sides of the same coin: heat/energy transfer and fluidics. We demonstrated interface-dependent physical phenomena inspired by vertically aligned nanostructures and confirmed the beneficial aspects of the interfaces for single-phase impinging-jet cooling. The principal factors governing the impinging-jet cooling scheme were presented and the feasibility of nano-inspired interfaces was demonstrated for possible local and overall heat transfer improvement. Interactivity of the nanostructures with fluidic boundary layer development could offer advantages in terms of quasi-fin effects and diminish macroscale friction loss. The exclusive quasi-fin effects as well as the stability of the viscous sublayer could be guaranteed when the thickness of viscous sublayers in the turbulent regime is much larger than the height of the employed nanostructures, so *δ*_*VSL*_*  ≫ l*_*fin*_. Since prevention of the friction-induced pressure drop was achieved by the disturbance-attenuated interfacial structures, the strategic nanostructures led to enhancement of local heat transfer coefficients in the wall jet region (at *x*/*W* = 6) of more than 21% without any significant macroscale pressure-drop increase, which is the common trade-off in heat transfer following implementation of macroscale surface roughening. For novel cooling schemes, nano-inspired interfacial modifications can be feasible with its fluidic and heat transfer advantages: control of the characteristic length of the structures is a key to defining the heat dissipation mode. Dimensional approaches and novel designs of *smart* nanostructure will give shape to possible breakthroughs in heat transfer and its optimization can be pursued in future work.

## Methods

### Fabrication of array-type local temperature measuring sensor

**Figure 1:**
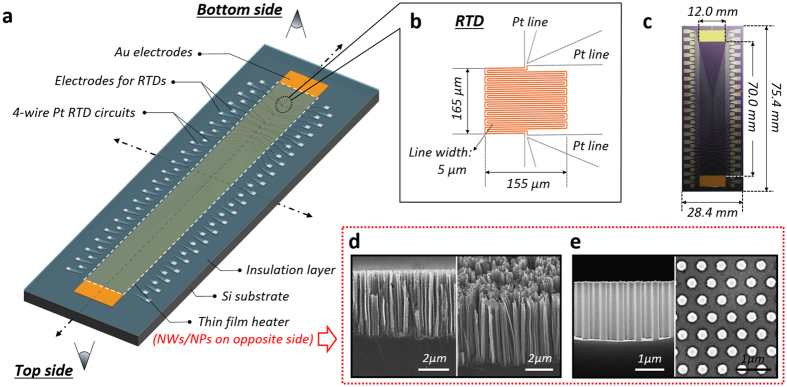
Local temperature measuring sensor and *in-situ* Si nanostructures synthesized on a target side. (**a**) Schematic of the sensor. (**b**) Enlarged view of a temperature-measuring resistance-temperature detector (RTD) with four-wire electric circuits. (**c**) Photograph of the completed sensor. (**d**) Vertical silicon nanowires (SiNWs), with an average height and diameter of 7 μm and 150 nm, respectively. (**e**) Regularly arranged silicon nanopillars (SiNPs) with a height of 2.5 μm and controlled diameter and pitch of 250 nm and 610 nm, respectively. The Si-nanostructures are synthesized just on the opposite side (Top side) of a thin film heater with an area of 70.0 × 12.0 mm^2^.

**Figure 2:**
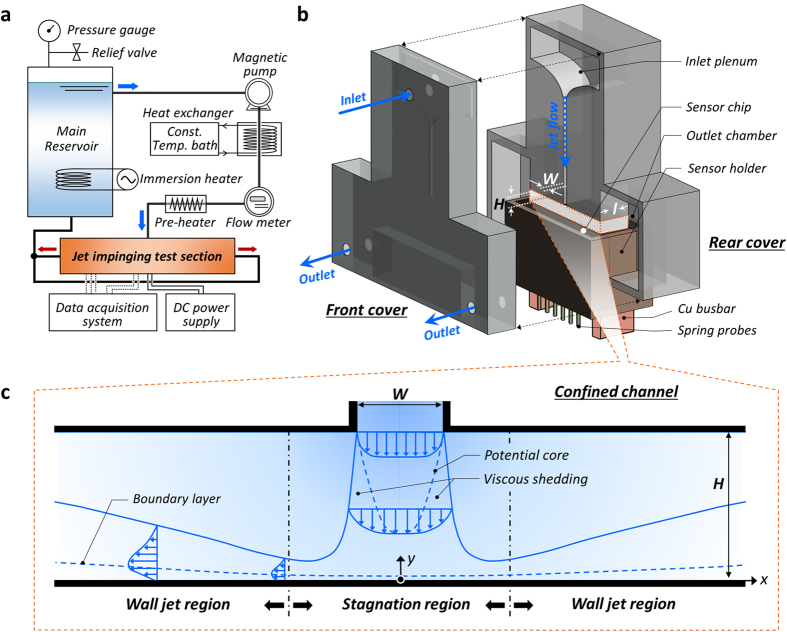
Schematic of the experimental apparatus for the evaluation of convective heat transfer with an impinging jet. (**a**) Closed-loop system for impinging jet heat transfer evaluation. (**b**) Test section for installing a local temperature measuring sensor with a confined impinging jet and symmetric exhaust flow. (**c**) Schematic of liquid impinging-jet cooling in a confined channel and sequential fluidic boundary formations in the stagnation region and the wall jet region.

**Figure 3:**
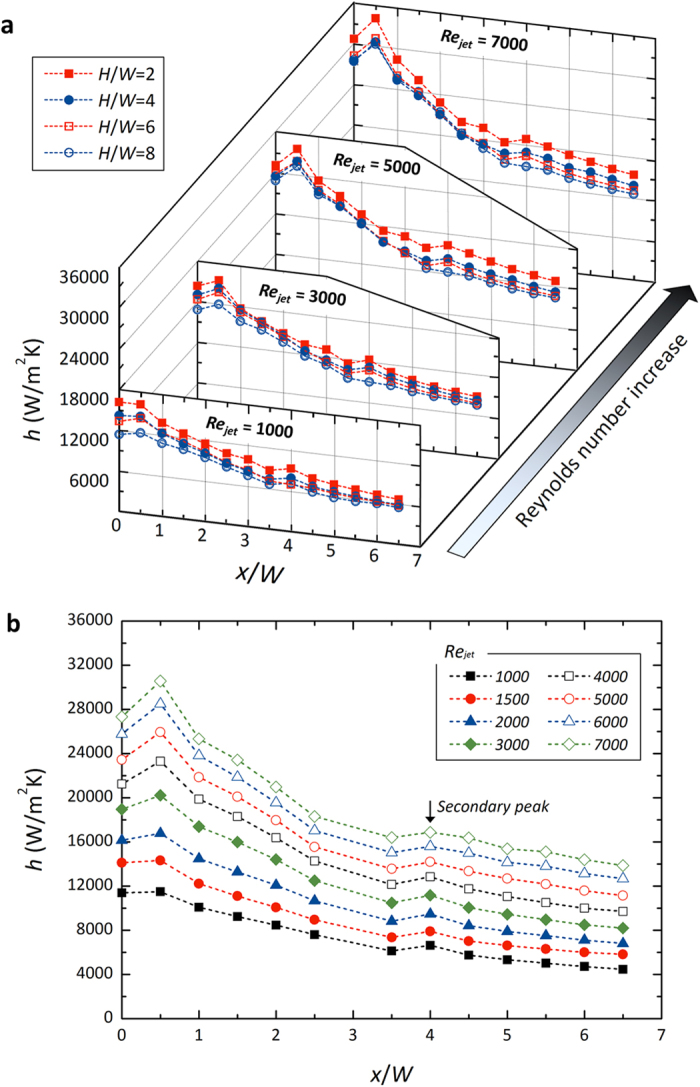
Local heat transfer coefficients on a plain surface. (**a**) Effects of channel height (*H*/*W*) and the inlet (jet) Reynolds number (*Re*_*jet*_). (**b**) Effects of *Re*_*jet*_ at *H*/*W* = 4.

**Figure 4:**
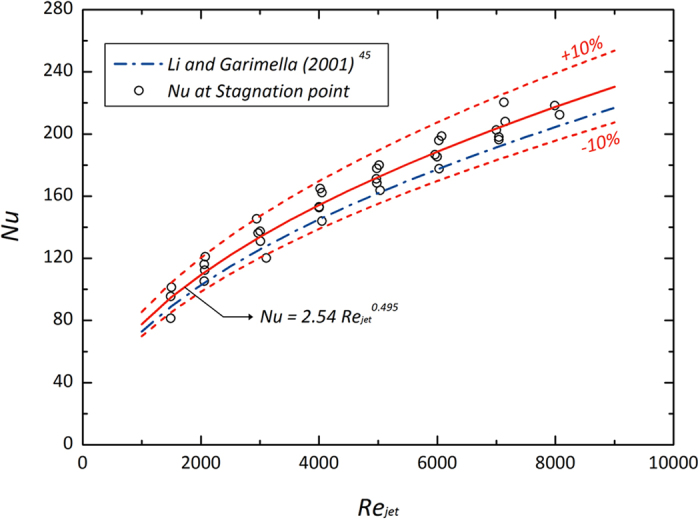
Convective local heat transfer at the stagnation point^45^.

**Figure 5:**
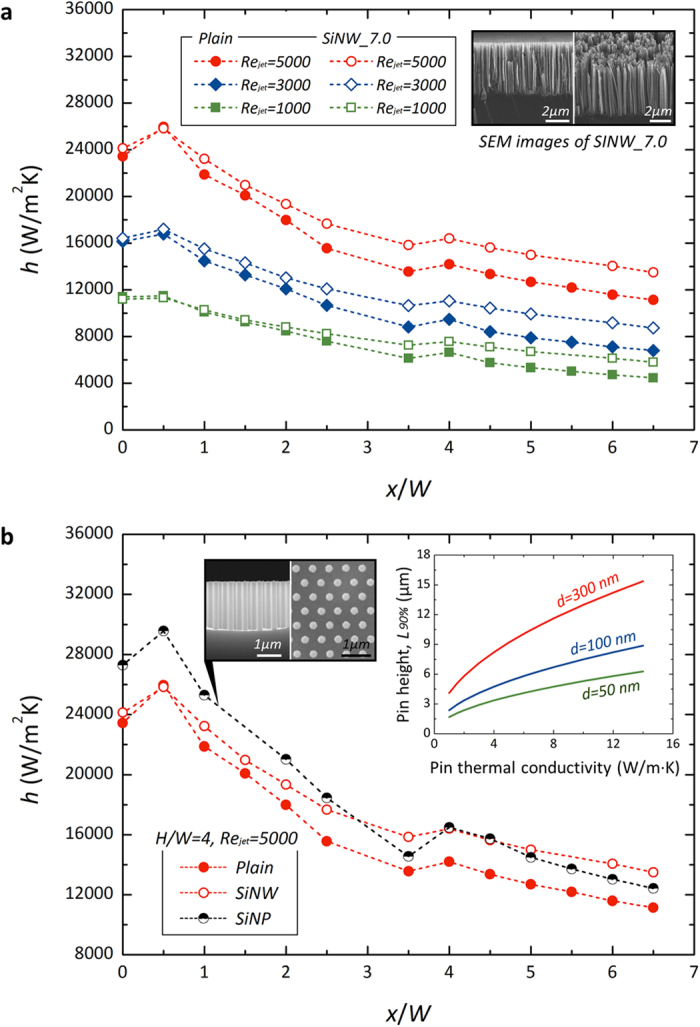
Nano-inspired surfaces for local heat transfer enhancement. (**a**) Effects of SiNWs with 7 micron-height at *H*/*W* = 4. (**b**) Comparison of SiNWs and SiNPs (pitch: 610 nm, diameter: 250 nm, and height: 2.5 μm) at *H*/*W* = 4 and *Re*_*jet*_* = *5000. Inset indicates effective length prediction for a conductive pin structure satisfying 90% heat transfer ratio.

**Figure 6:**
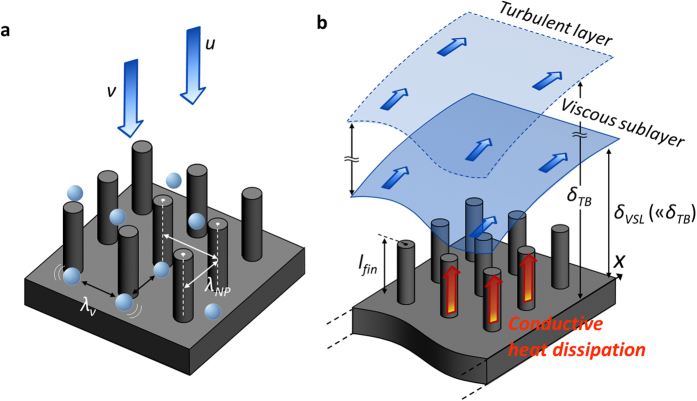
Interactivity of nanostructures for heat transfer enhancements. (**a**) Effect of viscous-governing liquid access over the stagnation area. (**b**) Quasi-fin effect through nanostructures and interactivity with fluidic boundary layers.

**Figure 7:**
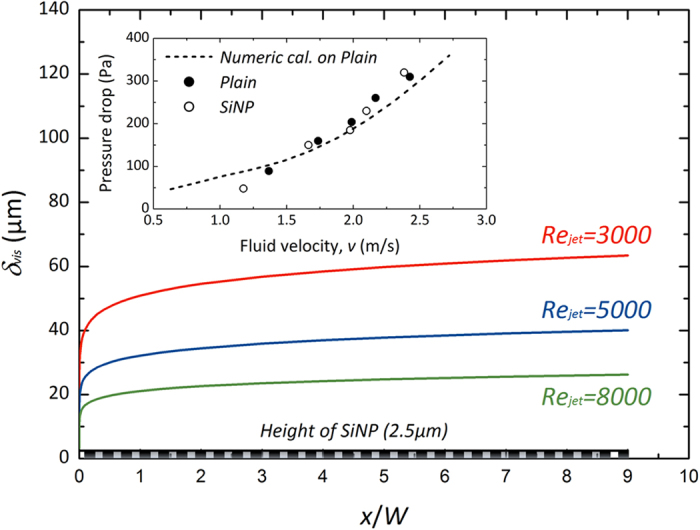
The predicted viscous sublayer thickness of *δ*_*VSL*_ along a horizontal plate according to *Re*_*jet*_. Inset presents measured pressure drop results from an untreated plain and SiNP surfaces.

The sensor is an integrated device comprising 20 four-wire resistance temperature detectors (RTDs) and a thin-film heater, fabricated on a 500 μm-thick silicon substrate (P-type, boron-doped, (100) orientation, resistivity of 1–10 Ω cm) with an area of 75.4 × 28.4 mm^2^. The top side of the substrate (Fig. 1a) is used as a target surface for introducing the impinging jet. RTDs and a heater are fabricated on the bottom side of the substrate. RTDs are made of platinum (Pt) and the resistance of each component is measured by the four-wire circuitry. The temperature-sensing part of each RTD (Fig. 1b) has a serpentine shape with a line width of 5 μm and a detecting area of 165 × 155 μm^2^. Its resistance is approximately 600 Ω at room temperature and varied linearly with temperature. The thin-film heater is an 8000 Å-thick indium tin oxide (ITO) layer. The heating area is 70 × 12 mm^2^, which has an equivalent width of 12 mm to the depth of the jet nozzle (*l*) and the width of the flow channel (Fig. 2b). There are 20 RTDs along the centerline, just below the heater. The completed sensor is installed on the sensor holder, which has circuit connectors of 80 spring probes and two copper bars on electrodes of the four-wire RTDs and the heater, respectively. The spring probes directly contact the electrodes of the RTDs and are connected with the data acquisition system through electric field-shielded cables. The copper bars are for current delivery, passing through the thin-film heater. As a thermal insulating layer, an air gap with 0.1 mm thickness is occupied between the heater of the sensor and the sensor holder to prevent direct conductive heat dissipation (See Supplementary Information for experimental setup for impinging jet, procedure, and data reduction).

### Nano-structured surface manipulation

*In-situ* Si-nanostructures are employed by the top-down metal-assisted chemical etching method^30^,^31^,^32^,^33^. Because the etching solution can damage the circuitry of the sensors, only the target surface on the top side of the sensor substrate is exposed to the solution and the remaining substrate is covered with a Teflon holder during synthesis. The exposed target area is cleaned with acetone and methanol in a sonication bath and is rinsed with DI water. Then, piranha (1:3 mixture of H_2_O_2_ and H_2_SO_4_ for 40 min) and buffered-oxide etching (dilute HF solution for 5 min) cleaning are progressed to remove organic residue and native oxide layers, respectively. For synthesizing SiNWs, the Si substrate is dipped into a solution of 0.005 M AgNO_3_ and 4.8 M HF for 1 min. This results in Ag^+^ ions coating the substrate surface due to galvanic displacement. After rinsing the substrate with DI water, the Ag^+^ ion-coated surface is dipped into the etching solution of a mixture, 0.1 M H_2_O_2_ and 4.8 M HF. The noble metal, such as Ag^+^ ions, reduces the potential between Si and H_2_O_2_/H_2_O oxidant and catalyzes local oxidation of the Si substrate just below the Ag ions. The oxidized portion is simultaneously etched away by the HF. This series of reactions leads to the formation of vertically aligned nanoscale structures on the substrate (Fig. 1d). The characteristic height of the nanowires is controlled by the etching time, and a 7-μm height is obtained with a 30-min etching process. SiNPs are fabricated with a pre-process of coating and shaping of nanospheres on the target surface^16^,^61^. The polystyrene (PS) nanospheres (*d* = 610 nm, Invitrogen) are closed-packed hexagonally in a monolayer on the air-water interface using the Langmuir-Blodgett method. The closed-packed monolayer is then transferred to the target surface and residual liquid is dried sufficiently by natural atmospheric convection. The packed PS nanospheres are diminished by reactive ion etching with O_2_ plasma from 610 nm to 250 nm while maintaining their adhesion on the substrate and arrangement. Here, the pitch of the SiNPs is defined as the initial diameter of PS nanospheres and the residual PS nanospheres, as a mask layer, define the diameter of the final SiNPs. A thin Au layer is deposited onto the PS coated substrate using E-beam evaporation and then PS nanospheres are removed with chloroform in an ultrasonic bath. The patterned Au film is a catalytic layer, similar to the role of the Ag ions used for SiNWs. The substrate is immersed in a mixture of 5 M HF and 0.5 M H_2_O_2_ to obtain hexagonally arranged nanopillar structures (Fig. 1e). After the etching process (500 s for 2.5 micron-high SiNPs), the Au film is removed with Au etchant.

### Morphology characterization

Morphology of the synthesized nanostructures is characterized by scanning electron microscopy (SEM) and post image processing. The morphology characterization and related data reduction are carried out using field-emission SEM (JSM-7001F, JEOL).

## Additional Information

**How to cite this article**: Kim, B. S. *et al*. Nano-inspired smart interfaces: fluidic interactivity and its impact on heat transfer. *Sci. Rep.* **7**, 45323; doi: 10.1038/srep45323 (2017).

**Publisher's note:** Springer Nature remains neutral with regard to jurisdictional claims in published maps and institutional affiliations.

## Supplementary Material

Supplementary Information
