# Supplementary material for: Nano-inspired smart interfaces: fluidic interactivity and its impact on heat transfer
Source: Sci Rep. 2017 Mar 27;7:45323. doi: 10.1038/srep45323 (PMC5366894; doi:10.1038/srep45323)
Supplement: Supplementary Information [file srep45323-s1.pdf]

Supplementary Information (Scientific Reports)

Nano-inspired smart interfaces: fluidic interactivity and its impact on heat transfer

Beom Seok Kim[1,‡], Byoung In Lee[2,‡], Namkyu Lee[3], Geehong Choi[3], Thomas Gemming[1], Hyung Hee Cho[3,*]

[1]IFW Dresden, P. O. Box 270116, 01171 Dresden, Germany

[2]Samsung Electronics Co., Ltd, Digital Appliance Business R&D team, Samsung-ro 129, 16677 Suwon, Korea

[3]Department of Mechanical Engineering, Yonsei University, Yonsei-ro 50, 03722 Seoul, Korea

[‡]These authors contributed equally to this work

[*]Corresponding author

Tel.: +82 2 2123 2828

Fax: +82 2 312 2159

E-mail: hhcho@yonsei.ac.kr

1. Convective heat transfer with impinging jet

1.1. Experimental setup: closed-loop system and test section

In the manuscript, **Fig. 2a** shows schematics of the experimental apparatus for a closed-loop channel and a test section for the installation of a local temperature measuring sensor. The closed-loop system consists of a stainless water reservoir, a degassing chamber, a plate-type heat exchanger, copper tubes, a magnetic pump, a flow meter, and an inverter to regulate water flow. The heat exchanger is connected with a constant temperature water bath and a rod-type pre-heater is also installed to accurately control fluid temperature. A 2-kW DC power supply is used to supply power to the heater. The test section (**Fig. 2b**) consists of a front cover, a rear cover, a core, a local temperature-measuring sensor, a sensor holder, and a holder aligner. The working fluid is supplied to a plenum, which is used as a settling chamber ahead of the inlet of a jet nozzle, at the upper region of the test section. The fluid flows along a fully developing region of a long nozzle neck, jet nozzle, and symmetric wall jet regions after jet impingement at a stagnation point and discharges towards opposite plenums connected to the outlets in the lower region of the test section. Herein, the straight fully developing region is sufficiently guaranteed for a restriction of any fluidic disturbance, with a length 15 times the nozzle width (W) for a stable velocity profile at the nozzle exit, which has a cross-section of 3.0 mm (width, W) ×12.0 mm (depth, l). The sidewalls of the channel are confined by the front and rear covers and the width of the channel is the same as the nozzle width. The height of the channel can be changed by adjusting the core components. The sensor holder is made of ceramic Macerite (thermal conductivity of 1.6 W/m·K) to prevent conductive heat loss and to maintain mechanical stability against thermal deformation. The other components, except for the sensor holder, are made of polycarbonate, which has high temperature stability above 150°C. Gas rejection valves are installed at each plenum to discharge dissolved gas before experiments. K-type thermocouples are mounted at the upper plenum and the downstream plenum to measure the respective inlet and outlet temperatures of the working fluid. Pressure taps are installed at the

fully developing region and the two wall jet regions, and are connected to a pressure transducer (PSCK-0050RCPG-B, Sensys Co.). Electrical signals are obtained by a data acquisition module (34970A, Agilent Technologies). For the acquisition of temperature-dependent resistance data and pressure drop data, we additionally use a high speed data sampling device (USB-6259, National Instruments).

1.2. Preconditioning and experimental procedure

Before the experiments, a degassing process is conducted by repeatedly boiling and condensing the working fluid. During degassing, the de-ionized (DI) water of the working fluid is boiled with an immersion heater in the main reservoir and the generated vapor is directed to an auxiliary chamber connected to the reservoir and then condensed. Degassed and condensed water is re-collected in the reservoir by gravity. After degassing, the temperature of the fluid is controlled at 60°C and this is kept constant as the jet inlet condition. The DC power supply is turned on and a heat flux of 20 W/cm^2 is supplied through the ITO thin film heater for heat transfer characterization. When a steady state condition is confirmed at the heat flux value, all data are acquired for more than 30 s through synchronization of the data-acquisition system. The data reported in this study are averaged for 30 s at the steady state condition. For the characterization, we accurately control the fluidic variable of the Reynolds number (*Re*) from laminar to turbulent regimes. The geometric variables regarding the jet nozzle and the channel dimensions of nozzle depth (l), nozzle width (W), channel height (H), and distance along a wall jet region from a stagnation point (x) are controlled and are expressed as normalized indicators for the results.

2. Data reduction and uncertainty analysis

2.1. Applied heat flux and local wall temperature

The heat flux applied to the thin-film heater is evaluated by measuring currents passing through the heater and the consequential voltage drop from the heater. The current (I) is measured by the voltage drop of a shunt (10 A-50 mV) inserted in the middle of the power circuitry and the voltage drop (V) is measured between the copper bars contacting the two opposite electrodes of the ITO heater. The heat flux q'' is expressed as $q'' = I \cdot V/A$, where A is the area of the film heater. The local wall temperature from each RTD is evaluated according to their individual pre-calibration, estimating the relationship between resistance and temperature. A heat flux is generated constantly from the film heater by Ohmic heating and then transferred conductively to its rear side, contacting the working fluid. We assume that the heat flux generated is transferred thoroughly towards the working fluid through 1-D conduction across the sensor substrate since it has a high thermal conductivity (k_{Si}) of 140 W/m·K with a layer thickness (t_{Si}) of 500 μm. According to the 1-D Fourier law of heat conduction, the local temperature at a local spot contacting the fluid, T_w, is deduced as $T_{RTD}-(t_{Si} \cdot q'')/k_{Si}$, where T_{RTD} is the temperature evaluated from a RTD[1–3]. The HTC is estimated based on Newton's law of cooling as $HTC = q''/(T_w-T_f)$, where T_f is the bulk fluid temperature[1,2]. The Nusselt number is expressed as $Nu = HTC \cdot D_h/k_f$, where D_h and k_f indicate the hydraulic diameter of the jet nozzle, and thermal conductivity of the working fluid, respectively.

2.2. Numerical modeling

To estimate the pressure drop of impinging jet experiments, we conduct numerical simulations using a commercial code, CFX 15.0 (ANSYS), which is widely used for simulating various fluidic phenomena. We consider the governing equations in steady state condition which consists of continuity and momentum, which can be expressed as follows[4]:

$$\nabla \cdot (\rho \vec{v}) = 0 \quad \text{(Eq.S1)}$$

$$\vec{v}(\nabla \cdot \vec{v}) = -\nabla\left(\frac{p}{\rho}\right) + (\mu + \mu_t)\nabla^2 \vec{v} \quad \text{(Eq.S2)}$$

where ρ, $\vec{v}$, p, μ, and μ_t are the fluid density, velocity, pressure, dynamic viscosity, and turbulent viscosity, respectively. To describe the turbulent fluidic phenomena due to impinging jets, we use the shear stress transport (SST) model, which has been more accurate than other turbulent models[5]. The number of tetra grids cells used in the simulation is more than 1700000 which is determined from the preliminary grid independent test. The working fluid is incompressible water, and the inlet condition is velocity inlet which is changed by the Reynolds number and the outlet condition is ambient pressure to reflect the experimental condition.

2.3. Uncertainty

An uncertainty analysis is performed based on the method presented by Kline and McClintock[6]. Quantitative uncertainty is estimated with a confidence level of 95% with the data pool obtained by repetitive experiments for each variable. The uncertainty is evaluated not only for the fundamental dimensions and measurements reflecting correlation-induced errors but also for the principal variables describing the results and demonstrations. The uncertainties regarding dimensions and related factors are as follows: 0.40% for dimension estimations in the devised sensor, 0.31% for the hydraulic diameter, 0.3 K for temperature measured through the conventional thermocouples, 1.9% for flow rate measurements, 1.6% for pressure measurement, and 2.12% for Re_{jet}. For the representative parameters defining the applied heat flux, local wall temperature (measured by the devised RTD), heat transfer coefficient and fluidic conditions, we consider the accumulation of propagated errors from each factor, which is physically subordinate to other variables. The errors on electric signals were computed by considering the accuracy of the measuring systems based on the

most recent calibration history. Herein, a conductive heat loss was calculated by additional simulation, and a total power loss (3.3%) is estimated by reflecting the conductive heat dissipation through a sensor substrate and the correlation error. The heat transfer characterization variables have uncertainties of 4.8% in applied heat flux, 4.9% in local wall temperature, 5.4% in *HTC*, and 5.4% in *Nu*.

References

1. Incropera, F. P. *Fundamentals of heat and mass transfer*, (John Wiley, 2007).
2. Kim, B. S. *et al.* Stable and uniform heat dissipation by nucleate-catalytic nanowires for boiling heat transfer. *Int. J. Heat Mass Transf.* **70,** 23-32 (2014).
3. Choi, G., Kim, B. S., Lee, H., Shin, S. & Cho, H. H. Jet impingement in a crossflow configuration: convective boiling and local heat transfer characteristics. *Int. J. Heat Fluid Flow* **50,** 378-385 (2014).
4. ANSYS CFX-Solver Theory Guide (ANSYS, 2013).
5. Zuckerman, N. & Lior, N. Jet impingement heat transfer: physics, correlations, and numerical modeling. *Advances in Heat Transfer* **39,** 565-631 (2006).
6. Kline, S. J. The purposes of uncertainty analysis. *J. Fluids Eng.* **107,** 153-160 (1985).
